# Analysis of Short Tandem Repeat Expansions in a Cohort of 12,496 Exomes from Patients with Neurological Diseases Reveals Variable Genotyping Rate Dependent on Exome Capture Kits

**DOI:** 10.3390/genes16020169

**Published:** 2025-01-28

**Authors:** Clarissa Rocca, David Murphy, Chris Clarkson, Matteo Zanovello, Delia Gagliardi, Queen Square Genomics, Rauan Kaiyrzhanov, Javeria Alvi, Reza Maroofian, Stephanie Efthymiou, Tipu Sultan, Jana Vandrovcova, James Polke, Robyn Labrum, Henry Houlden, Arianna Tucci

**Affiliations:** 1 Department of Neuromuscular Diseases, UCL Institute of Neurology, London WC1N 3BG, UK; clarissa.rocca.19@ucl.ac.uk (C.R.); david.murphy@ucl.ac.uk (D.M.); m.zanovello@ucl.ac.uk (M.Z.); d.gagliardi@ucl.ac.uk (D.G.); rauan.kaiyrzhanov.14@ucl.ac.uk (R.K.); r.maroofian@ucl.ac.uk (R.M.); s.efthymiou@ucl.ac.uk (S.E.); jana.vandrovcova@ucl.ac.uk (J.V.); h.houlden@ucl.ac.uk (H.H.); 2 William Harvey Research Institute, Queen Mary University of London, London EC1M 6BQ, UK; c.clarkson@ucl.ac.uk; 3 Department of Pathophysiology and Transplantation (DEPT), University of Milan, 20122 Milan, Italy; 4 Department of Paediatric Neurology, The Children’s Hospital and the University of Child Health Sciences, Lahore 54000, Punjab, Pakistan; drjaveriarazaalvi@gmail.com (J.A.); tipusultanmalik@hotmail.com (T.S.); 5 Neurogenetics Unit, National Hospital for Neurology and Neurosurgery, London WC1N 3BG, UK; j.polke@ucl.ac.uk (J.P.); robyn.labrum@gosh.nhs.uk (R.L.); 6 Department of Neurodegenerative Disease, UCL Queen Square Institute of Neurology, University College London, London WC1E 6BT, UK

**Keywords:** short tandem repeat, ExpansionHunter, short read sequencing, exome sequencing, repeat expansion diseases, myotonic dystrophy, spinocerebellar ataxia, Huntington disease, repeat expansion disease

## Abstract

Background/Objectives: Short tandem repeat expansions are the most common cause of inherited neurological diseases. These disorders are clinically and genetically heterogeneous, such as in myotonic dystrophy and spinocerebellar ataxia, and they are caused by different repeat motifs in different genomic locations. Major advances in bioinformatic tools used to detect repeat expansions from short read sequencing data in the last few years have led to the implementation of these workflows into next generation sequencing pipelines in healthcare. Here, we aimed to evaluate the clinical utility of analysing repeat expansions through exome sequencing in a large cohort of genetically undiagnosed patients with neurological disorders. Methods: We here analyse 27 disease-causing DNA repeats found in the coding, intronic and untranslated regions in 12,496 exomes in patients with a range of neurogenetic conditions. Results: We identified—and validated by polymerase chain reaction—29 repeat expansions across a range of loci, 48% (*n* = 14) of which were diagnostic. We then analysed the genotyping performance across all repeat loci and found that, despite high coverage in most repeats in coding regions, some loci had low genotyping rates, such as those that cause spinocerebellar ataxia 2 (*ATXN2,* 0.1–8.4%) and Huntington disease (*HTT*, 0.2–58.2%), depending on the capture kit. Conversely, while most intronic repeats were not genotyped, we found a high genotyping rate in the intronic locus that causes spinocerebellar ataxia 36 (*NOP56,* 30.1–98.3%) and in the one that causes myotonic dystrophy type 1 (*DMPK,* myotonic dystrophy type 1). Conclusions: We show that the key factors that influence the genotyping rate of repeat expansion loci analysis are the sequencing read length and exome capture kit. These results provide important information about the performance of exome sequencing as a genetic test for repeat expansion disorders.

## 1. Introduction

Many patients with suspected genetic conditions remain undiagnosed after standard genetic testing [1,2]. Repeat expansions are a major cause of inherited neurological disease, with over 60 diseases described to date, caused by the same underlying mechanism: the expansion of short repetitive DNA sequences (1–6 bp) within their respective genes. Clinically, they present as neurodevelopmental, neuromuscular and neurodegenerative disorders. Among the most common repeat expansion diseases (REDs) are fragile X syndrome (GCC repeat in 5′ untranslated regions (UTR) of the *FMR1* gene), myotonic dystrophy (CTG repeat in 3′ UTR of the *DMPK* gene), spinocerebellar ataxias (many caused by exonic CAG repeats in their respective genes) and cerebellar ataxia, neuropathy and vestibular areflexia syndrome (CANVAS; AAGGG repeat expansion in the intron of *RFC1*).

One of the main limitations of current short-read DNA sequencing technologies until recently was the inability to detect repeat expansions, which can reach thousands of units repeats [3]. In fact, the most common sequencing read-length of these technologies varies from about 75 to 150 base pairs. In the last few years, bioinformatic workflows have been developed to detect small and large repeat expansions from short read genome and exome sequencing [4,5].

Here, we aimed to investigate the clinical utility of repeat expansion analysis using exome sequencing from a large cohort of genetically undiagnosed patients with neurological diseases. We combined bioinformatics analysis with experimental validation and then analysed the factors that determine locus coverage and genotyping quality at each locus assessed.

To achieve this, we used ExpansionHunter3.1.2 (EH) [6] to analyse 12,496 selected exomes from the UCL Institute of Neurology’s neurogenetic database, which consists of samples collected over the years through worldwide collaborations. This diverse collection includes both patients with undiagnosed neurological disorders and their family members, providing a valuable resource for studying repeat expansions.

## 2. Methods

### 2.1. Cohort

A total of 12,496 exomes were analysed from the UCL Institute of Neurology. This database is composed of data from patients collected over the years as a result of collaborations across the globe. Consequently, DNA samples have been sequenced on different platforms, at different sequencing read lengths, and using different exome enrichment kits (Supplementary Table S1). Exome sequencing data are processed in a homogenous manner. Paired-end reads are aligned to the GRCh38 human reference using the Burrows–Wheeler Aligner BWA-MEM [7] and processed using the GATK (version 4.1.4.0) best practices to create BAM files [8]. Sex and ethnicity were derived using peddy (version 0.4.3) [9].

### 2.2. Repeat Genotyping

**Figure 1:**
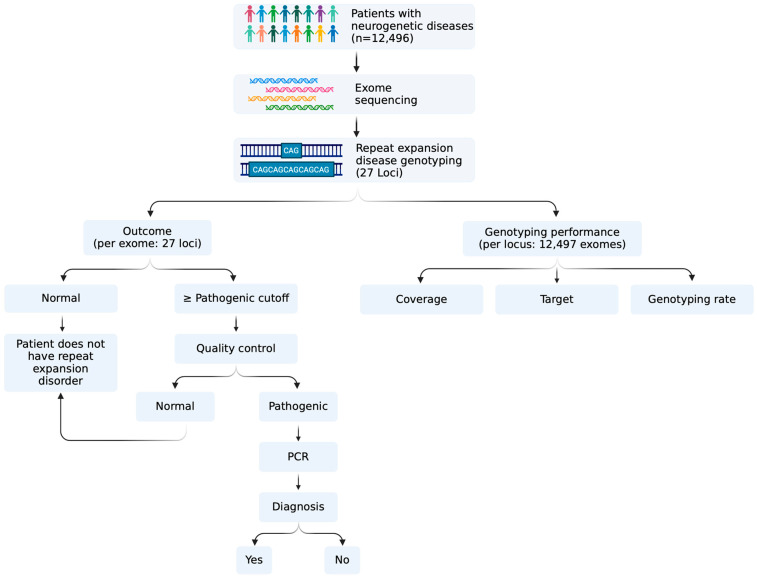
Schematic overview of the study workflow.

Repeat genotyping was performed using EH software version 3.1.2, a well-established tool for detecting disease-causing repeat expansions [6]. We used genomic coordinates provided in the EH variant catalogue for calling repeats in the human genome version 38 (b38). Pileup plots were generated using GraphAlignmentViewer (https://github.com/Illumina/GraphAlignmentViewer, accessed on 10 December 2024) on the BAM files generated by EH. Locus coverage (LC) was calculated using samtools depth to obtain the number of reads from the samples’ BAM files. EH coverage was extracted from the LC field in the EH-generated VCF files. A schematic representation of the workflow used in this study is provided in Figure 1.

### 2.3. Visual Inspection

The visual inspection of sequencing reads used by EH to predict a genotype greatly improves specificity and allows for the identification of sequence interruptions. The level of accuracy in detecting repeat expansions from next generation sequencing is affected by sequencing read length: if the pathogenic cut-off of a given disease-causing repeat is larger than the sequencing read length, normal alleles cannot be accurately distinguished from expanded alleles. Therefore, we classified each predicted expanded call into “pass”, “borderline” or “fail”, taking into account for each call the number and quality of reads supporting each genotype by visual inspection, the sequencing read length used to generate the corresponding exome and the presence of interruptions. Calls were defined as “pass” if the corresponding exome was generated with sequencing reads longer than the pathogenic cut-off of the predicted expanded repeat, and if the genotype was supported by at least two high-quality reads or a large number (>10) of reads reaching the maximum expansion; “borderline” calls were defined as supported by one high quality read in an exome with a read length bigger than the pathogenic cutoff for the specific locus, or calls supported by two or more high-quality reads in an exome with a read length shorter than the pathogenic cut-off for their specific locus. Pileups that did not meet any of the above criteria were defined as “fail”.

### 2.4. PCR Validation

In order to validate the presence of a repeat expansion identified by EH, a combination of repeat-primed PCR (RP-PCR), fragment analysis and gel electrophoresis was used, depending on the gene. RP-PCR was used to qualitatively assess for the presence of the repeated sequence in each analysed locus. Details of PCR-based methods are provided in the supplementary materials.

## 3. Results

**Figure 2:**
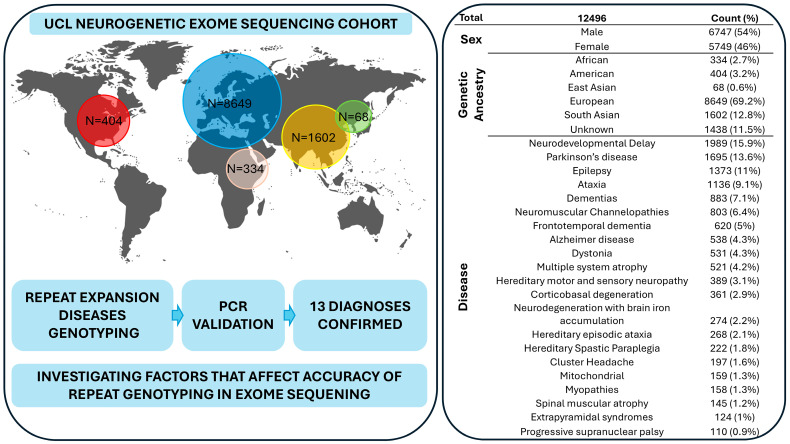
Cohort overview and study design. The map illustrates the global distribution of 12,496 cases included in the cohort, with participant numbers represented by coloured circles: Europe (N = 8649, blue), East Asia (N = 1602, yellow), Africa (N = 404, red), America (N = 334, dark red), and South Asia (N = 68, green). The right panel provides the demographic information and diagnostic categories included in the analysis. The study design is summarised in the blue boxes at the bottom.

Over the last several years, the Queen Square Genomics Group at University College London has collected exome sequencing and clinical data from patients with suspected neurogenetic conditions, both nationally and internationally. This large database allows researchers to analyse single nucleotide variants and small indels across cases or validate findings and has successfully led to new gene identification, improving the diagnostic yield for patients. However, repeat expansion analysis has not been performed thus far. To test the utility of this analysis and improve diagnostics for these patients, we ran Expansion Hunter on data from 12,496 individuals in this cohort. This cohort comprises data from patients with a range of neurological disorders, including neurodevelopmental, neurodegenerative and neuromuscular conditions (Figure 2).

Though exome sequencing is designed to capture the protein-coding genes and, in some cases, UTRs of the genome, it has been observed that over half of sequences routinely originate outside of coding exons [10], noncoding DNA sequences in exon-flanking DNA parts, and promoter regions. We therefore selected 27 repeat expansion disease (RED) loci known to cause neurological diseases, regardless of their genomic location (that is coding regions, UTRs and intron regions) (Table 1, Supplementary Table S2).

**Figure 3:**
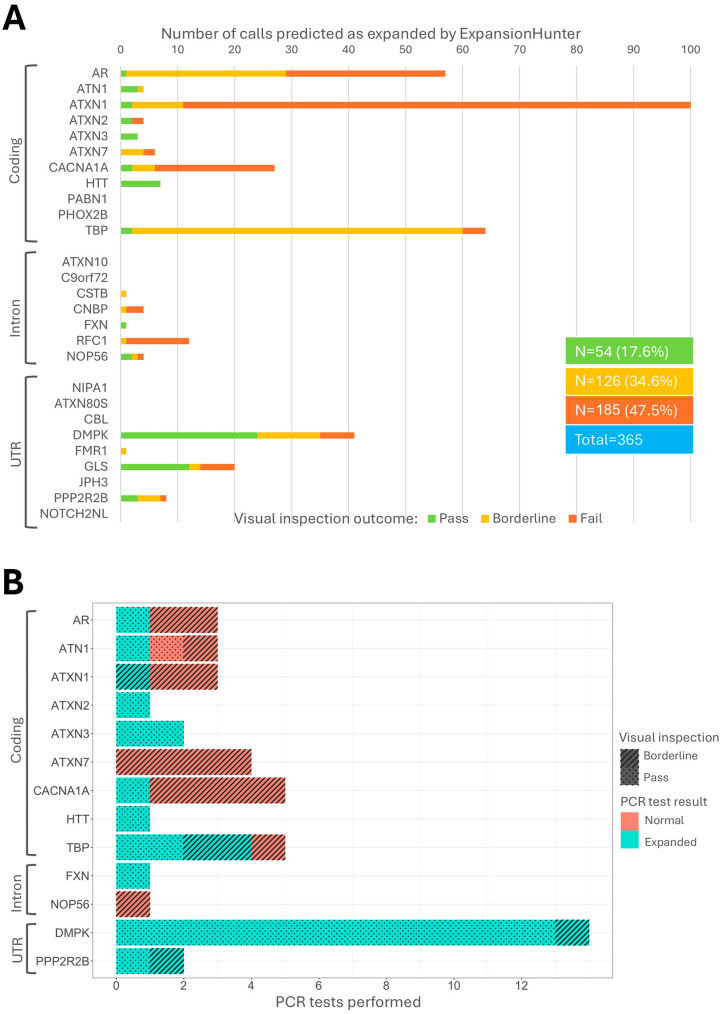
Total number of repeat expansions identified by EH, visual inspection and PCR validation. (**A**) 365 repeat expansions identified by EH with the visual inspection outcome. Loci are divided into three groups: coding, intron and UTR. Green bars represent calls that passed visual inspection, yellow bars are for calls that were categorised in the “borderline” group and red bars indicate samples that failed visual inspection. Loci that do not have a bar next to them did not have any expanded calls predicted by EH. (**B**) The outcome of PCR-tested samples. The light blue bars indicate samples that tested positive for PCR, while the pink bars represent samples that tested negative. Stripes indicate cases that were in the visual inspection “Pass” category, whereas dots represent cases that were “borderline” after visual inspection.

EH identified a total of 365 (2.91%) expanded alleles in the pathogenic or premutation range across 18 loci (Figure 3, Supplementary Table S3). Following visual inspection, 50.7% (*n* = 185) of the total predicted expanded calls were classified as “fail” and discarded from further analyses. Of those remaining, the largest number of calls with predicted expansions were the ones in *DMPK* (myotonic dystrophy), *GLS* (global developmental delay, progressive ataxia and elevated glutamine) and *HTT* (Huntington disease). The loci with the largest number of “fail” were *RFC1* (*n* = 11, 92%), followed by *ATXN1* (*n* = 89, 89%) (Figure 3A). Three additional calls in the pathogenic range in *ATXN1* were discarded due to the presence of CAT sequence interruptions (Supplementary Figure S1). As only biallelic mutations in GLS cause disease, we searched for predicted damaging variants in the entire genomic region in each exome with a heterozygous repeat expansion. No potentially pathogenic variants were identified.

We then retrieved all available DNA samples from all individuals with a repeat classified as “pass” or “borderline” by visual inspection and performed PCR-based tests of the putatively expanded repeats. Of all the samples tested (*n* = 45), 29 were confirmed as expanded (24 “pass” and 5 “borderline”) (Figure 3B, Supplementary Table S3).

After reviewing the clinical data of patients with a PCR-validated expansion, 14 patients were confirmed to a have a diagnosis of repeat expansion disorder, with myotonic dystrophy type 1 being the most common (Table 2). Notably, four of these cases were part of the same family (Table S4). As for the remaining exomes with validated expansions in the pathogenic range, either clinical data was insufficient to confirm the diagnosis, the expansion was in the reduced penetrance range or the patient had not yet reached the typical age at onset, which is typically associated with smaller repeats (Table S4). Here, we present a detailed description of a family with SCA3.

### Clinical Description of the SCA3 Case

**Figure 4:**
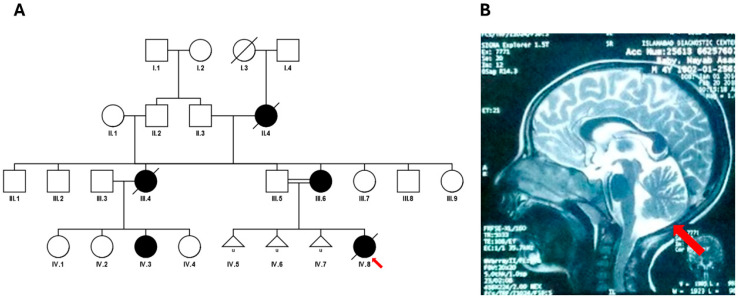
Pedigree of SCA3 family and MRI scan of proband. The red arrow shows the proband. (**A**) Square = male; circle = female; black filled symbol = affected individual; white symbols = unaffected individuals; diagonal line = deceased individual. Double lines indicate consanguinity. (**B**) MRI scan of patient IV.8. The red arrow indicates cerebellar atrophy.

Of note, we describe the SCA3 case, harbouring 23/>81 repeats. The proband, a Pakistani paediatric patient born to consanguineous parents (Figure 4A), presented with speech regression, muscle wasting, and motor axonal polyneuropathy. She was developmentally age-appropriate until 3.5 years of age, when she began experiencing frequent falls, walking difficulty with swaying, and progressive spasticity of the arms and legs. Over time, she developed difficulty chewing and swallowing, became completely bedbound, and showed nystagmus after 5 years of age. Developmental regression over the last six months of life led to complete loss of speech, vision and independent ambulation. Neurological examination revealed increased muscle tone, brisk deep tendon reflexes, and muscle wasting. Systemic examination was unremarkable. The metabolic workup, including creatine phosphokinase (CPK), lactate dehydrogenase (LDH), and ceruloplasmin levels, was normal. MRI of the brain showed cerebellar atrophy (Figure 4B), and fundoscopy findings were normal. Electromyography and nerve conduction studies confirmed motor axonal polyneuropathy. The patient passed away at 6 years of age due to a respiratory infection leading to respiratory failure. The family history included three spontaneous abortions, and the proband was the only living child.

We then analysed exome data from 20 patients in the cohort that had been previously tested for repeat expansions as part of their standard diagnostic workup, comprising 60 PCR results, for a total of 120 alleles from 13 loci. EH correctly predicted 119/119 normal alleles, missing one monoallelic *FXN* expansion (Supplementary Figure S2) that was located in an intronic region not covered by the exome capture kit used (Nextera DNA focused).

**Figure 5:**
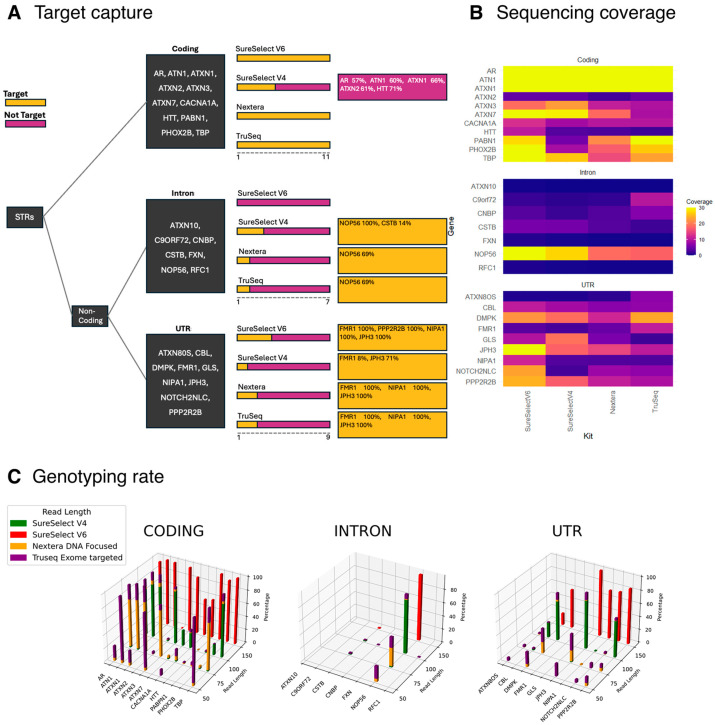
Targeted loci and coverage according to the four most used exome sequencing kits in this cohort. (**A**) The RED loci are categorised based on their genomic location: coding, intron and UTR. Target (purple): the specific region of the gene is targeted by the exome kit. Not target (yellow): the region of interest is not covered by the exome kit. The percentage indicates how much of the region is not covered. For example, in *ATN1*, 60% of the region of interest is not covered by the SureSelect V4 kit. When not specified, the percentage of target or not target is 0%. The exome sequencing kits are represented by different bars: SureSelect V6, SureSelect V4, Nextera and TruSeq. The dashed lines under each group indicate the total number of RED loci analysed in each category: 12 coding, 7 intronic and 8 UTRs. (**B**) Heatmap showing the coverage of the analysed RED loci across different genomic regions. Coverage is represented by the number of sequencing reads mapping to each locus, as indicated by the colour scale. (**C**) 3D plots of the genotyping rate for EH-generated calls by read length and sequencing kit. The three plots show EH calls in coding, intron and UTR loci. In each plot, calls are divided by locus and read length. The four different colours represent the different exome capture kits used.

In order to gain insights into the performance of repeat expansion genotyping using exome sequencing, we then compared the coverage and genotyping performance (indicated as the proportion of each predicted genotype classified by EH as “pass”) of RED regions across the four most commonly used exome sequencing kits in our cohort, namely SureSelect V6, SureSelect V4, Truseq Exome Targeted and Nextera DNA Focused. Interestingly, this analysis revealed that the coverage and the genotyping quality for coding RED loci were good (>20×) across all genes, with the exception of *CACNA1A*, *ATXN2* and *HTT,* which cause spinocerebellar ataxia 2, spinocerebellar ataxia 6 and Huntington disease, respectively. For *ATXN2*, the percentage of “pass” calls was lower compared to the other coding loci (Supplementary Table S3). We observed that the EH calculated coverage was consistently lower than the coverage derived from BAM files, which may be attributed to decreased targeting of these regions by the sequencing kits (Supplementary Figure S3). In contrast, for *HTT* and *CACNA1A*, the percentage of “pass” calls increased as the sequencing read length increased. Interestingly, EH was able to detect RED loci in genomic regions not targeted by sequencing kits. For example, the intronic repeat in *NOP56,* which causes spinocerebellar ataxia 36, had an average of 20× coverage, depending on the capture kit; repeats in UTRs, such as *JPH3*, *DMPK*, *NOTCH2NLC* and *PPP2R2B* (which cause Huntington disease-like 2, myotonic dystrophy type 1, neuronal intranuclear inclusion disease and spinocerebellar ataxia 12), were also captured and their genotypes predicted (Figure 5, Supplementary Table S2).

Moreover, we looked at the percentage of “pass” genotypes in coding, intron and UTR expansions separately, categorising by read length. Our findings indicate that longer read lengths correlated with a higher percentage of “pass” calls (Figure 5C) regardless of their genomic location. This trend is consistent across the four most commonly used sequencing kits (Supplementary Tables S5–S7).

## 4. Discussion

In this study, we analysed repeat expansions in patients with undiagnosed neurological diseases using bioinformatic analysis followed by experimental validation. Our methodology involved analysing a large cohort of exomes from our in-house database, consisting of 12,469 samples from patients with neurological diseases. We identified a total of 365 expanded alleles in the pathogenic or premutation range; however, following a quality check by visual inspection, 50.7% (*n* = 185) were discarded and 49.3% were considered as putatively expanded. Following the PCR validation of available DNA samples from putatively expanded calls (*n* = 45), we were able to confirm an expansion in 29 patients, 14 of which were confirmed diagnostically. These data indicate that analysis of REDs by exome sequencing requires extensive quality checks and that orthogonal tests have a high validation rate for calls that are supported by at least two spanning reads or ten flanking reads. The exome database we worked on had a large number of samples that were received in the form of sequencing data, and DNA was not available. However, the validation rate was high considering the minimum number of reads.

The findings presented here suggest that EH is able to differentiate between expanded and non-expanded alleles at any analysed locus that is covered by the exome kit used if the sequencing read length is bigger than the pathogenic cutoff. In cases where the presence of an interruption determines the pathogenicity of the expansion, such as those in *ATXN1*, visual inspection is essential for differentiating positive and negative cases. These findings broadly replicate previous studies that tested the accuracy and utility of RED analysis in exome sequencing in patients with neurological disorders [5,11,12].

The analysis of the genotyping rate of individual RED loci revealed one important limitation of repeat expansion detection by exome sequencing: common coding RED loci—such as *ATXN2* and *HTT,* which cause spinocerebellar ataxia 2 and Huntington disease, respectively—may not be sufficiently covered depending on the exome capture kit used: the maximum genotyping rate was 8.8% for *ATXN2* targeted by Truseq Exome, and 58.2% for *HTT* by SureSelect V6. Conversely, UTR and intronic loci, such as *DMPK* and *NOP56* (myotonic dystrophy type 1 and spinocerebellar ataxia 36), have higher genotyping rates across all exome capture kits analysed. These results suggest that EH is able to identify pathogenic repeat expansions, even at those loci that might be not targeted by conventional exome sequencing kits, such as Agilent’s SureSelect V6. Importantly, we note that this pattern has emerged in previous studies: in the study by Yoon et al. (12), which employed SureSelectV5 and V6, *ATXN2* was excluded by the analysis due to low coverage, and in Van der Sander’s study, an *ATXN2* expansion was missed by the same kit (5). Conversely, in the study by Mereaux (11), which employed Twist capture, all loci analysed were sufficiently covered, including *NOP56*.

In conclusion, our study demonstrates the utility of bioinformatic analysis with experimental validation for identifying and validating repeat expansions in exomes from patients with neurological diseases, and that the utility of this analysis largely depends on the capture kits used: some show extremely low coverage of coding repeats and high coverage of some intronic and UTR repeats. The high validation rate achieved highlights the importance of quality checks in improving the accuracy of expansion calling.

These findings contribute to our understanding of repeat expansion-associated neurological disorders and provide valuable insights for future diagnoses.

## Figures and Tables

**Table 1:** Repeat expansion loci analysed in this study. This table summarises the loci analysed, with their associated diseases, repeat structures, the cutoffs used to discriminate between expanded and non-expanded alleles in repeats and base pairs, as well as genomic locations.

Gene	Disease	Repeat Motif	Cutoff (Repeat Units)	Cutoff in bp	Genomic Location
*AR*	Spinal and bulbar muscular atrophy	CAG	36	108	Coding
*ATN1*	Dentatorubral–pallidoluysian atrophy	CAG	35	105	Coding
*ATXN10*	Spinocerebellar ataxia 10	ATTCT	33	165	Intron
*ATXN1*	Spinocerebellar ataxia 1	CAG	39	117	Coding
*ATXN2*	Spinocerebellar ataxia 2	CAG	32	96	Coding
*ATXN3*	Spinocerebellar ataxia 3	CAG	45	135	Coding
*PHOX2B*	Congenital central hypoventilation syndrome	GCN	NA	NA	Coding
*ATXN7*	Spinocerebellar ataxia 8	CAG	33	99	Coding
*ATXN80S*	Spinocerebellar ataxia 8	CAG	40	120	3′UTR
*C9orf72*	Frontotemporal dementia and/or amyotrophic lateral sclerosis	GGGGCC	30	180	Intron
*CACNA1A*	Spinocerebellar ataxia 6	CAG	19	57	Coding
*CNBP*	Myotonic dystrophy 2	CCTG	27	108	Intron
*DMPK*	Myotonic dystrophy 1	CTG	36	108	3′UTR
*FMR1*	FMR1-related disorders	CGG	55	165	5′UTR
*FXN*	Friedreich ataxia	GAA	34	102	Intron
*HTT*	Huntington disease	CAG	35	105	Coding
*JPH3*	Huntington disease-like 2	CTG	49	147	Exon
*NOP56*	Spinocerebellar ataxia 36	GGCCTG	15	90	Intron
*PPP2R2B*	Spinocerebellar ataxia 12	CAG	33	99	5′UTR
*TBP*	Spinocerebellar ataxia 17	CAG	43	129	Coding
*NIPA1*	Hereditary Spastic Paraplegia type 6	GCG	NA	NA	5′UTR
*NOTCH2NL*	Neuronal intranuclear inclusion disease	GGC	55	165	5′UTR
*RFC1*	Cerebellar ataxia, neuropathy, and vestibular areflexia syndrome	AAGGG	0	5	Intron
*PABN1*	Oculopharyngeal muscular dystrophy	GCN	NA	NA	Coding
*CSTB*	Progressive myoclonic epilepsy 1A	CCCCGCCCCGCG	4	48	Intron
*GLS*	Global developmental delay, progressive ataxia, and elevated glutamine	GCA	30	90	5′UTR

**Table 2:** Clinical description of 14 patients with a confirmed diagnosis of repeat expansion diseases among all patients tested.

Gene	Repeat GT	Sex	Ethnicity	Clinical Details
*ATN1*	16/>80	Female	South Asian	Epilepsy with developmental delays.
*ATXN2*	23/38	Male	European	Slowly progressive cerebellar ataxia syndrome with evidence of weakness in the lower limbs and mild spastic increased tone. No extrapyramidal signs.
*ATXN3*	23/>81	Female	South Asian	Speech regression, wasting of muscles, motor axonal polyneuropathy and seizures.
*ATXN3*	27/60	Male	American	Ataxia.
*DMPK*	8/60	Female	South Asian	Myotonic dystrophy as well as motor and sensory neuropathy.
*DMPK*	12/>150	Male	South Asian	Hereditary peripheral neuropathy and myotonic features; 46 y at examination.
*DMPK*	9/>150	Male	Unknown	Affected brother. Myotonia atrophica and hereditary motor and sensory neuropathy; 40 y at examination.
*DMPK*	12/60	Male	Unknown	Hereditary peripheral neuropathy; 42 y at examination.
*DMPK*	9/>150	Male	European	Myotonia atrophica and hereditary motor and sensory neuropathy; 50 y at examination.
*DMPK*	8/>150	Male	European	Myotonia atrophica and hereditary motor and sensory neuropathy. Affected nephew; 35 y at examination.
*DMPK*	9/>150	Female	European	Myotonia atrophica and hereditary motor and sensory neuropathy. Affected niece; 29 y at examination.
*HTT*	29/53	Female	European	Ataxia, hyperreflexia, chorea, neurodevelopmental delays, no extra-ocular or sphincter involvement, cerebellar and brain stem atrophy on MRI.
*TBP*	38/57	Male	Unknown	Familial SCA, early death.

## Data Availability

Dataset available on request from the authors.

## Supplementary Materials

The following supporting information can be downloaded at: https://www.mdpi.com/article/10.3390/genes16020169/s1, Figure S1. Pileup plot of *AXN1* with interruptions and *RFC1* with non-pathogenic configuration. The top panel shows the pileup plot of an *ATXN1* case with an A interruption that was predicted as expanded by EH. The bottom panel shows an *RFC1* expanded call carrying a non-pathogenic configuration “AAAAG”. Figure S2. Correlation between EH predicted allele size and PCR observed size. Each data point represents the intersection of a predicted and observed allele size, with the size of the dot indicating the frequency of that specific combination. Figure S3. Comparison between EH coverage and number of reads from BAM files in *ATXN2.* EH consistently gives a lower coverage rates to the *ATXN2* locus across the four most commonly used sequencing kits in our cohort. Table S1. Exome capture kit and sequencing read lengths. Different sequencing read lengths, and exome enrichment kits. Table S2. Coverage by repeat and by capture kit. Table S3. Outcome of Visual inspection. Detailed breakdown of visual inspection outcome of individual genes. Samples available for testing are reported in the Samples available column. Samples that were confirmed expanded by PCR test are further divided by their visual inspection outcome. Table S4. Summary of all PCR validated expansions. Details of all PCR validated expansions with associated clinical details and diagnostic outcome. Table S5. Genotyping rate per coding loci, by genomic region and sequencing kit. Table S6. Genotyping rate per intronic loci, by genomic region and sequencing kit. Table S7. Genotyping rate per UTR loci, by genomic region and sequencing kit.

## References

[1] Marwaha S., Knowles J.W., Ashley E.A. (2022). A guide for the diagnosis of rare and undiagnosed disease: Beyond the exome. Genome Med..

[2] Record C.J., Reilly M.M. (2024). Lessons and pitfalls of whole genome sequencing. Pract. Neurol..

[3] Bansal V., Boucher C. (2019). Sequencing Technologies and Analyses: Where Have We Been and Where Are We Going?. IScience.

[4] Ibañez K., Polke J., Hagelstrom R.T., Dolzhenko E., Pasko D., Thomas E.R.A., Daugherty C.L., Kasperaviciute D., Smith R.K., WGS for Neurological Diseases Group (2022). Whole genome sequencing for the diagnosis of neurological repeat expansion disorders in the UK: A retrospective diagnostic accuracy and prospective clinical validation study. Lancet Neurol..

[5] Van der Sanden B.P.G.H., Corominas J., De Groot M., Pennings M., Meijer R.P.P., Verbeek N., Van de Warrenburg B., Schouten M., Yntema G.H., Vissers E.L.M.L. (2021). Systematic analysis of short tandem repeats in 38,095 exomes provides an additional diagnostic yield. Genet. Med..

[6] Dolzhenko E., Deshpande V., Schlesinger F., Krusche P., Petrovski R., Chen S., Emig-Agius D., Gross A., Narzisi G., Bowman B. (2019). Expansion Hunter: A sequence-graph-based tool to analyze variation in short tandem repeat regions. Bioinformatics.

[7] Li H., Durbin R. (2009). Fast and accurate short read alignment with Burrows–Wheeler transform. Bioinformatics.

[8] Van der Auwera G.A., Carneiro M.O., Hartl C., Poplin R., Del Angel G., Levy-Moonshine A., Jordan T., Shakir K., Roazen D., Thibault J. (2013). From FastQ data to high confidence variant calls: The Genome Analysis Toolkit best practices pipeline. Curr. Protoc. Bioinform..

[9] Pedersen B.S., Quinlan A.R. (2017). Who’s who? Detecting and resolving sample anomalies in human DNA sequencing studies with peddy. Am. J. Hum. Genet..

[10] Samuels D.C., Han L., Li J., Quanghu S., Clark T.A., Shyr Y., Guo Y. (2013). Finding the lost treasures in exome sequencing data. Trends Genet..

[11] Méreaux J.L., Davoine C.S., Coutelier M., Guillot-Noël L., Castrioto A., Charles P., Coarelli G., Ewenczyk C., Klebe S., Heinzmann A. (2023). Fast and reliable detection of repeat expansions in spinocerebellar ataxia using exomes. J. Med. Genet..

[12] Yoon J.G., Lee S., Cho J., Kim N., Kim S., Kim M.J., Kim S.Y., Moon J., Chae J.-H. (2024). Diagnostic uplift through the implementation of short tandem repeat analysis using exome sequencing. Eur. J. Hum. Genet..

