# Supplementary material for: Analysis of Short Tandem Repeat Expansions in a Cohort of 12,496 Exomes from Patients with Neurological Diseases Reveals Variable Genotyping Rate Dependent on Exome Capture Kits
Source: Genes (Basel). 2025 Jan 28;16(2):169. doi: 10.3390/genes16020169 (PMC11855749; doi:10.3390/genes16020169)
Supplement: Supplementary file 1 [file genes-16-00169-s001.zip › genes-3400153-supplementary.pdf]

## Supplementary Figures

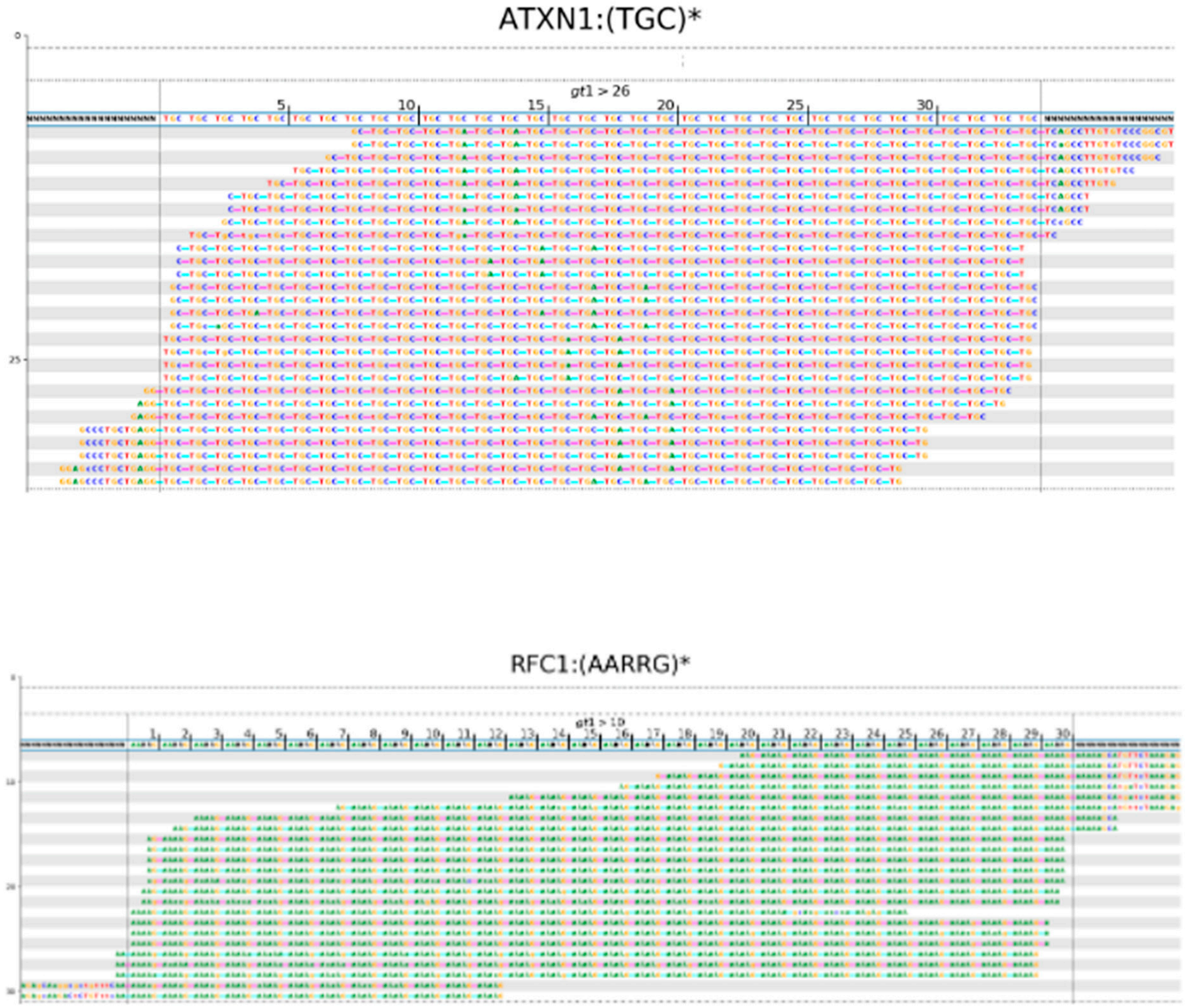


**Figure S1. Pileup plot of *AXN1* with interruptions and *RFC1* with non-pathogenic configuration.** The top panel shows the pileup plot of an *ATXN1* case with an A interruption that was predicted as expanded by EH. The bottom panel shows an *RFC1* expanded call carrying a non-pathogenic configuration "AAAAG".

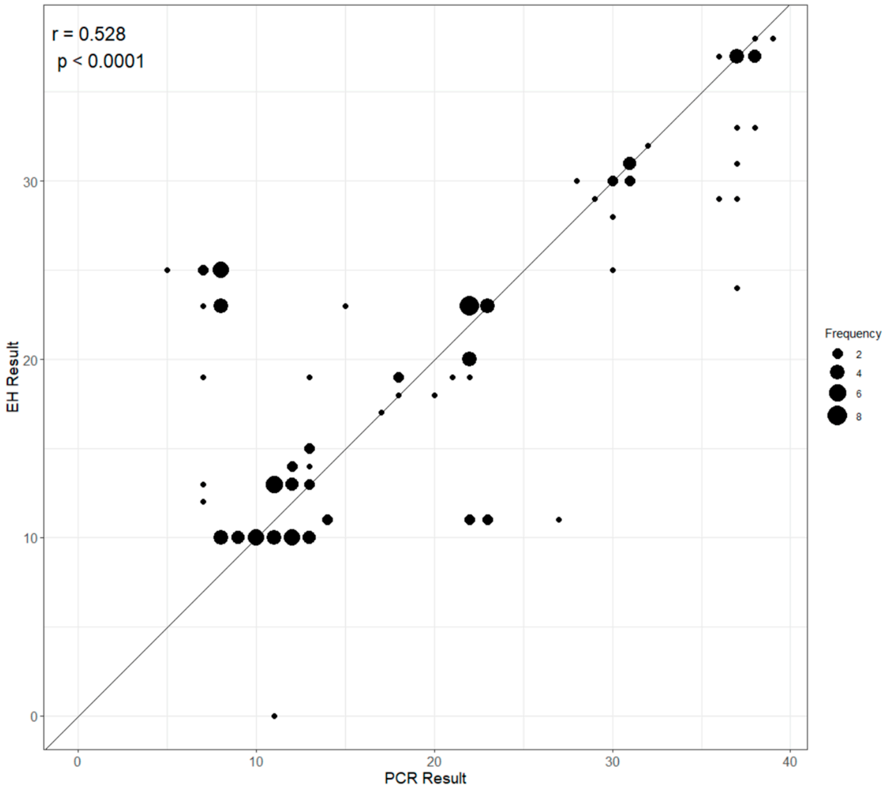


**Figure S2. Correlation between EH predicted allele size and PCR observed size.** Each data point represents the intersection of a predicted and observed allele size, with the size of the dot indicating the frequency of that specific combination.

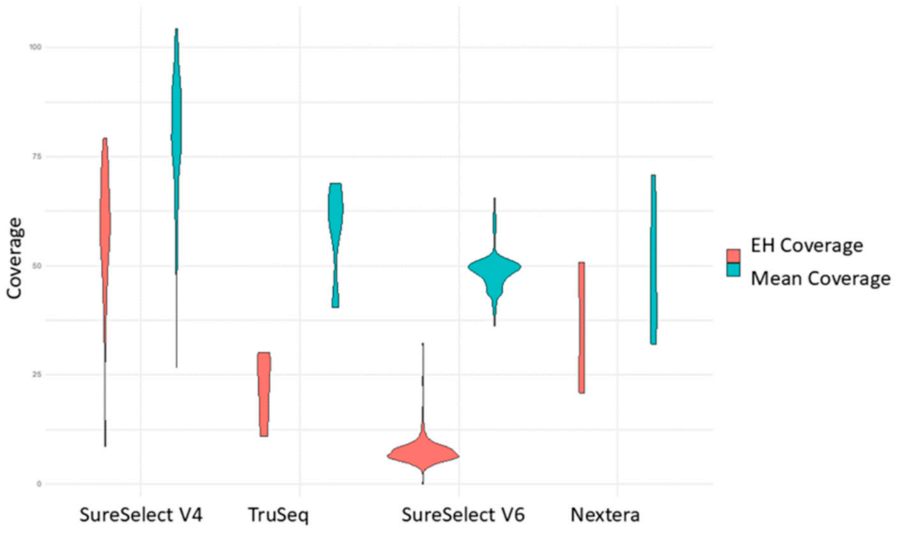


**Figure S3. Comparison between EH coverage and number of reads from BAM files in *ATXN2*.** EH consistently gives a lower coverage rates to the *ATXN2* locus across the four most commonly used sequencing kits in our cohort.

## Supplementary Tables

**Table S1. Exome capture kit and sequencing read lengths.** Different sequencing read lengths, and exome enrichment kits.

| Sequencing Kit | Read length (bp) | Number of exomes (%) |
|---|---|---|
| **SureSelect V6** | 150 | 2302 (18.4) |
| **SureSelect V4** | 75 | 9 (0.07) |
| | 100 | 3757 (30.1) |
| **Nextera DNA Focused** | 50 | 1 (0.01) |
| | 75 | 386 (3.1) |
| | 100 | 293 (2.3) |
| **Truseq Exome Targeted** | 50 | 21 (0.2) |
| | 75 | 123 (1) |
| | 100 | 580 (4.6) |
| **Other** | 50 | 81 (0.6) |
| | 75 | 939 (7.5) |
| | 100 | 2322 (18.6) |
| | 125 | 847 (6.8) |
| | 150 | 835 (6.7) |
| **Total** | | **12496** |

**Table S2.** Coverage by repeat and by capture kit.

| Gene | Full Name | Genomic Position (GRCh38) | Genomic Location | SureSelect V6 (Mean, Median, Range) | | SureSelect V4 (Mean, Median, Range) | | Nextera DNA Focused (Mean, Median, Range) | | Truseq Exome targeted (Mean, Median, Range) | |
|---|---|---|---|---|---|---|---|---|---|---|---|
| | | | | EH Coverage | BAM Coverage | EH Coverage | BAM Coverage | EH Coverage | BAM Coverage | EH Coverage | BAM Coverage |
| *AR* | Androgen Receptor | X:67545316-67545385 | Coding | (78, 67.8, 23.6-519) | 100% (25.6, 23, 0-168) | (58.9, 49.6, 0-217.9) | 43.48% (29, 27, 260-147) | (36, 29.3, 1.6-477.2) | 100% (25.5, 16, 0-550) | (73.4, 64.3, 0.5-280.6) | 100% (39.9, 31, 0-251) |
| *ATN1* | Atrophin 1 | 12:6936716-6936773 | Coding | (117.7, 119.7, 16.8-496.5) | 100% (122.2, 129, 0-505) | (67.6, 65, 0-218.8) | 40.35% (88.2, 83, 0-513) | (33.2, 28.7, 3-331.1) | 100% (43.1, 20, 0-896) | (48.2, 45.5, 2.1-281.8) | 100% (59.7, 55, 0-398) |
| *ATXN 1* | Ataxin 1 | 6:16327633-16327723 | Coding | (103.5, 105.1, 21.3-485.7) | 100% (35.2, 37, 0-129) | (103.1, 100.1, 0-295.5) | 34.44% (70.6, 71, 0-242) | (61.3, 50.7, 4.9-367) | 100% (53, 38, 0-1012) | (48.2, 45.5, 2.1-281.8) | 100% (76.3, 64, 0-432) |
| *ATXN 2* | Ataxin 2 | 12:111598949-111599018 | Coding | (4.4, 4.4, 0-24.5) | 100% (8.6, 5, 0-126) | (4.4, 4.4, 0-24.5) | 39.13% (20.1, 13, 0-267) | (2.2, 1.3, 0-50.3) | 100% (11.3, 7, 0-366) | (4.6, 3.7, 0-42.4) | 100% (18.6, 14, 0-196) |
| *ATXN 3* | Ataxin 3 | 14:92071009-92071042 | Coding | (18.6, 18.6, 3.3-47.2) | 100% (36.4, 36, 2-117) | (22.9, 22.3, 0-73.7) | 100% (86.6, 85, 0-345) | (10.9, 7.7, 0.1-76.4) | 100% (38.5, 23, 0-282) | (9.4, 8.4, 0.7-99.5) | 100% (63.6, 58, 0-808) |
| *ATXN 7* | Ataxin 7 | 3:63912684-63912726 | Coding | (37.2, 36.5, 10.9-137) | 100% (14.7, 13, 0-117) | (29.2, 28.4, 6.3-89.5) | 100% (9.1, 8, 0-66) | (18.3, 16.6, 1.7-155.4) | 100% (7.37, 3, 0-174) | (9, 8.2, 0-46.6) | 100% (0.02, 0, 0-3) |

| | | | | | | | | | | | |
|---|---|---|---|---|---|---|---|---|---|---|---|
| *ATXN10* | Ataxin 10 | 22:45795354-45795424 | Intron | (0.3, 0.2, 0-2.7) | 0% (0.2, 0, 0-7) | (0.2, 0.2, 0-4.4) | 0% (0.1, 0, 0-5) | (0.2, 0.1, 0-6.5) | 0% (0.2, 0, 0-7) | (0.2, 0.1, 0-9.6) | 0% (0.2, 0, 0-25) |
| *ATXN8OS* | Ataxin 8 | 13:70139353-70139428 | 3'UTR | (0.7, 0.6, 0-4.6) | 0% (3.6, 3, 0-33) | (0.7, 0.6, 0-3.8) | 0% (7, 6, 0-55) | (1.3, 0.4, 0-18.1) | 0% (7.2, 3, 0-155) | (6.9, 5.7, 0-27.5) | 0% (8.9, 8, 0-60) |
| *C9orf72* | Chromosome 9 open reading frame 72 | 9:27573528-27573546 | Intron | (1.7, 1.6, 0-12.7) | 0% (0.9, 0, 0-14) | (1.3, 1.2, 0-4) | 0% (0.7, 0, 0-7) | (1.4, 0.6, 0-27.8) | 0% (0.7, 0, 0-11) | (10, 10, 0-40.2) | 0% (0.9, 1, 0-9) |
| *CACNA1A* | Calcium channel, voltage-dependent, p/q type, alpha-1a subunit | 19:13207858-13207897 | Coding | (12.7, 12.2, 0.6-81.2) | 100% (7.5, 7, 0-54) | (8.6, 8.1, 0-31.5) | 100% (13.1, 12, 0-81) | (10, 7.4, 0.1-133.6) | 100% (5.3, 4, 0-122) | (10, 9, 0-126.2) | 100% (4.9, 3, 0-78) |
| *CBL* | Casitas B-lineage lymphoma proto-oncogene | 11:119206289-119206322 | 5'UTR | (10.6, 9.6, 2.1-65.7) | 0% (3.8, 3, 0-23) | (8.6, 7.8, 0-37.7) | 0% (9.6, 8, 0-75) | (6.8, 5.8, 0.1-130.4) | 0% (3.1, 1, 0-33) | (7.4, 5.3, 0-87.1) | 0% (2.9, 0, 0-48) |
| *CNBP* | Cchc-type zinc finger nucleic acid-binding protein | 3:129172576-129172732 | Intron | (3.4, 3.3, 0.6-9.7) | 0% (1.5, 1, 0-280) | (1.8, 1.7, 0.2-4.4) | 0% (1.2, 1, 0-26) | (3.2, 2.2, 0-37.3) | 0% (0.6, 0, 0-12) | (6.4, 5.6, 0.3-26.8) | 0% (0.9, 0, 0-20) |
| *CSTB* | Cystatin B | 21:43776443-43776479 | Intron | (5.7, 5.6, 0-22.4) | 0% (3.4, 3, 0-25) | (5.4, 5.1, 0-19.7) | 13.89% (5.4, 5, 0-36) | (3.4, 2.7, 0-65.7) | 0% (1.9, 1, 0-27) | (2.1, 1.5, 0-34.3) | 0% (1.4, 0, 0-27) |
| *DMPK* | Dystrophia myotonica protein kinase | 19:45770204-45770264 | 3'UTR | (22.1, 21.8, 3.3-126.3) | 0% (5.9, 5, 0-68) | (18.7, 17.9, 0-51.2) | 0% (9.1, 7, 0-118) | (12, 9.7, 0.2-133.1) | 0% (6, 3, 0-191) | (23.7, 23.1, 0-121) | 0% (16.3, 13, 0-100) |
| *FMR1* | Fragile x messenger ribonucleoprotein 1 | X:147912050-147912110 | 5'UTR | (3.7, 3.3, 0.2-21.7) | 100% (2.7, 2, 0-54) | (2.7, 2.4, 0-12.1) | 8.33% (2.1, 1, 0-33) | (4.6, 3, 0-165.5) | 100% (0.7, 0, 0-53) | (10.7, 9, 0.1-79) | 100% (0.1, 0, 0-9) |
| *FXN* | Frataxin | 9:69037261-69037304 | Intron | (0.9, 0.8, 0-3.5) | 0% (0.3, 0, 0-7) | (0.5, 0.5, 0-3.9) | 0% (0.2, 0, 0-8) | (0.3, 0.2, 0-4.8) | 0% (0.3, 0, 0-12) | (0.3, 0.2, 0-14.8) | 0% (0.2, 0, 0-15) |
| *GLS* | Glutaminase | 2:190880872-190880920 | 5'UTR | (9.5, 9.3, 0.5-58.8) | 0% (7.5, 7, 0-57) | (19, 18.4, 1.1-64.1) | 0% (48.1, 47, 0-333) | (5.9, 4.8, 0-108.8) | 0% (7, 4, 0-339) | (2.1, 1.6, 0-20.4) | 0% (7.3, 5, 0-63) |
| *HTT* | Huntingtin | 4:3074876-3074966 | Coding | (10.4, 10.9, 0-46.7) | 100% (30.1, 28, 0-200) | (3.4, 3.2, 0.1-12.5) | 28.89% (26.9, 24, 0-170) | (2.9, 2.3, 0-58.8) | 100% (14.8, 9, 0-676) | (2, 1.5, 0-29.8) | 100% (8.5, 5, 0-71) |
| *JPH3* | Junctophilin 3 | 16:87604287-87604329 | 3'UTR | (40.5, 40, 11.3-169.9) | 100% (79, 79, 10-245) | (17.7, 17.2, 0-49.7) | 71.43% (36.2, 35, 0-159) | (14.8, 11.7, 0.6-93.1) | 100% (34.9, 31, 0-504) | (10.4, 9, 0-48.6) | 100% (13.1, 10, 0-153) |
| *NIPA1* | NIPA magnesium transporter 1 | 15:22786677-22786701 | 5'UTR | (11.9, 11.9, 0-35.3) | 100% (78.9, 77, 0-273) | (3.3, 3.1, 0-11.2) | 0% (17.6, 17, 0-145) | (3.1, 2.3, 0-80.8) | 100% (4.9, 2, 0-183) | (3.1, 2.6, 0-40.1) | 100% (0.03, 0, 0-6) |
| *NOTCH2NLC* | Notch2 n-terminal-like c | 1:149390802-149390841 | 5'UTR | (23.5, 24.2, 0-165.9) | 0% (33.7, 32, 0-337) | (2.5, 2.4, 0-11) | 0% (13, 12, 0-79) | (8, 5.6, 0-69.2) | 0% (9.6, 5, 0-111) | (7.2, 5.2, 0-44.1) | 0% (13, 4, 0-177) |
| *NOP56* | NOP56 ribonucleoprotein | 20:2652733-2652775 | Intron | (38.4, 35.1, 14.4-184.7) | 0% (24.1, 24, 0-145) | (27.3, 26.4, 0-78.8) | 100% (38.8, 37, 0-218) | (18.6, 15, 1.4-120.8) | 69.05% (26.5, 23, 0-430) | (17.6, 14.9, 0.1-241.1) | 69.05% (18.8, 13, 0-129) |

| | | | | | | | | | | | |
|---|---|---|---|---|---|---|---|---|---|---|---|
| *PABN1* | polyadenylate-binding protein nuclear 1 | 14:23321472-23321490 | Coding | (27.6, 26.1, 5.2-182.6) | 100% (51.7, 48, 0-357) | (5.5, 5.2, 0-17.7) | 100% (10.5, 10, 0-68) | (21.3, 18, 1.4-301) | 100% (12.2, 4, 0-121) | (51.9, 52.1, 0-200.5) | 100% (4.3, 3, 0-35) |
| *PHOX2B* | Paired-like homeobox 2B | 4:41745972-41746032 | Coding | (30.1, 28.5, 4.9-164.6) | 100% (20.9, 17, 0-255) | (8.8, 8.6, 0.2-24.2) | 100% (8.9, 8, 0-61) | (17, 14.5, 1-253.2) | 100% (9.5, 3, 0-107) | (26.4, 25.4, 0.4-112.1) | 100% (1.6, 0, 0.42) |
| *PPP2R2B* | Protein phosphatase 2 regulatory subunit B | 5:146878727-146878757 | 5'UTR | (24.6, 22.9, 9.2-125.4) | 100% (11.3, 11, 0-40) | (17, 16.6, 0-47.6) | 0% (9.66, 9, 0-74) | (10.9, 8.8, 0.6-69.9) | 0% (8.7, 6, 0-98) | (9.2, 8.5, 0.6-87.6) | 0% (10.7, 7, 0-61) |
| *RFC1* | Replication factor C subunit 1 | 4:39348424-39348479 | Intron | (0.6, 0.6, 0-3.8) | 0% (0.2, 0, 0-9) | (0.4, 0.3, 0-4.9) | 0% (0.1, 0, 0-6) | (0.3, 0.2, 0-5.9) | 0% (0.5, 0, 0-13) | (0.3, 0.2, 0-13.2) | 0% (0.3, 0, 0-9) |
| *TBP* | TATA box binding protein | 6:170561906-170562017 | Coding | (35, 35.4, 5-86) | 100% (59.8, 61, 1-215) | (26.3, 25.8, 0-84.3) | 100% (91.3, 91, 0-362) | (15.3, 13.9, 0.6-80) | 100% (53.1, 29, 0-555) | (23.3, 21, 0.6-176.9) | 100% (57.8, 54, 0-485) |

**Table S3. Outcome of Visual inspection.** Detailed breakdown of visual inspection outcome of individual genes. Samples available for testing are reported in the Samples available column. Samples that were confirmed expanded by PCR test are further divided by their visual inspection outcome.

| Gene | Premutation Range | EH Expanded Calls (number of calls) | Visual Inspection | | | Samples available | Tot PCR positives (yes, borderline) |
|---|---|---|---|---|---|---|---|
| | | | Pass | Borderline | Fail | | |
| *AR** | 36-37 | 57 | 1 (1.79%) | 28 (50%) | 28 (48.21%) | 3 | 1 (1, 0) |
| *ATN1* | 35-47 | 4 | 3 (75%) | 1 (25%) | 0 (0%) | 3 | 1 (1, 0) |
| *ATXN1* | 38-44 | 100 | 2 (2%) | 9 (9%) | 89 (89%) | 3 | 1 (0, 1) |
| *ATXN2* | 33-34 | 4 | 2 (50%) | 0 (0%) | 2 (50%) | 1 | 1 (1, 0) |
| *ATXN3* | 45-59 | 3 | 3 (100%) | 0 (0%) | 0 (0%) | 2 | 2 (2, 0) |
| *ATXN7* | 28-33 | 6 | 0 (0%) | 4 (66.67%) | 2 (33.33%) | 4 | 0 |
| *ATXN10* | NA | 0 | NA | NA | NA | NA | NA |
| *ATXN80S* | NA | 0 | NA | NA | NA | NA | NA |
| *C9orf72* | 25-60 | 0 | NA | NA | NA | NA | NA |
| *CACNA1A* | 18-20 | 27 | 2 (7.41%) | 4 (14.81%) | 21 (77.78%) | 5 | 1 (1, 0) |
| *CBL* | NA | 0 | NA | NA | NA | NA | NA |
| *CNBP* | 27-29 | 4 | 0 (0%) | 1 (25%) | 3 (75%) | 0 | 0 |
| *CSTB* | 12-17 | 1 | 0 (0%) | 1 (100%) | 0 (0%) | 0 | 0 |
| *DMPK* | 35-49 | 42 | 24 (57.14%) | 11 (26.19%) | 7 (16.67%) | 14 | 14 (13, 1) |

| | | | | | | | |
|---|---|---|---|---|---|---|---|
| *FMR1* | 55-200 | 1 | 0 (0%) | 1 (100%) | 0 (0%) | 0 | 0 |
| *FXN* | 34-65 | 1 | 1 (100%) | 0 (0%) | 0 (0%) | 1 | 1 (1, 0) |
| *GLS*** | NA | 20 | 12 (60%) | 2 (10%) | 6 (30%) | 0 | 0 |
| *HTT* | 27-35 | 7 | 7 (100%) | 0 (0%) | 0 (0%) | 1 | 1 (1, 0) |
| *JPH3* | 29-39 | 0 | NA | NA | NA | NA | NA |
| *NIPA1* | NA | 0 | NA | NA | NA | NA | NA |
| *NOTCH2NLC* | NA | 0 | NA | NA | NA | NA | NA |
| *NOP56* | NA | 4 | 2 (50%) | 1 (25%) | 1 (25%) | 1 | 0 |
| *PABN1* | NA | 0 | NA | NA | NA | NA | NA |
| *PHOX2B* | 21-23 | 0 | NA | NA | NA | NA | NA |
| *PPP2R2B* | 31-50 | 8 | 3 (37.5%) | 4 (50%) | 1 (12.5%) | 2 | 2 (1, 1) |
| *RFC1* | NA | 12 | 0 (0%) | 1 (8.33%) | 11 (91.67% ) | 0 | 0 |
| *TBP* | 41-48 | 64 | 2 (3.13%) | 58 (90.63%) | 4 (6.25%) | 5 | 4 (2, 2) |
| *Total* | | 365 | 64 (17.53%) | 126 (34.52%) | 175 (47.95%) | 45 (25 Pass, 20 Borderline) | 29 (24, 5) |

*Males only. **The second allele was further analysed to exclude the presence of a second pathogenic variant.

**Table S4. Summary of all PCR validated expansions.** Details of all PCR validated expansions with associated clinical details and diagnostic outcome.

| Gene | LogID | Repeat GT | Sex | Ethicity | Disease Category | Details | Diagnosis | Comments |
|---|---|---|---|---|---|---|---|---|
| *AR* | Pt1 | 38 | Male | European | Episodic Ataxia | Episodic Ataxia. | No | Insufficient details |
| *AR* | Pt2 | 42 | Male | South Asian | Neurodevelopmental delay | Father of proband with complex movement disorder | No | Insufficient details |
| *ATN1* | Pt3 | 16/>80 | Female | South Asian | Epilepsy | Proband. Epilepsy with Developmental Delay | **YES** | |
| *ATXN2* | Pt4 | 23/38 | Male | European | Dementia | Slowly progressive cerebellar syndrome with evidence of weakness in lower limbs and mild spastic inc tone. No extrapiramidal signs | **YES** | |
| *ATXN3* | Pt5 | 23/>81 | Female | South Asian | Epilepsy | Speech regression, wasting of muscles, motor axonal polyneuropathy. | **YES** | Patient described in the main text and figure 3 |
| *ATXN3* | Pt6 | 27/60 | Male | American | Ataxia | Ataxia. Participant has withdrawn from the study. | **YES** | |
| *CACNA1A* | Pt7 | 13/20 | Male | South Asian | Epilepsy | Reduced Penetrance - Father of proband with seizures. | No | Reduced Penetrance |
| *DMPK* | Pt8 | 8/60 | Female | South Asian | Hereditary motor and sensory neuropathy | Myotinic Dystrophy, unsolved. | **YES** | |

| | | | | | | | | |
|---|---|---|---|---|---|---|---|---|
| *DMPK* | Pt9 | 12/>150 | Female | European | Epilepsy | Proband; epilepsy (seizures onset at 6 months old), MRI: porencephalic cyst. No consanguineity, no family history | No | Clinical diagnosis of myotonic dystrophy not confirmed, possibly due to age of proband below the age of onset |
| *DMPK* | Pt10 | 12/64 | Male | European | Hereditary motor and sensory neuropathy | Severe cerebellar ataxia with axonal neuropathy. | No | Proband does not have features of DM1, possibly due to age being earlier than the age of onset |
| *DMPK* | Pt11 | 12/>150 | Male | South Asian | Hereditary motor and sensory neuropathy | Hereditary Peripheral Neuropathy and myotonic features. 46yo at examination. | **YES** | |
| *DMPK* | Pt12 | 12/>150 | Male | European | Neurodevelopmental delay | Proband. Epilepsy, no speech, hydrocephaly. Consanguineity, no FH. | No | Clinical diagnosis of myotonic dystrophy not confirmed, possibly due to age of proband below the age of onset |
| *DMPK* | Pt13 | 9/>150 | Male | European | Hereditary motor and sensory neuropathy | Myotonia atrophica and neuropathy. 40y at examination | **YES** | Related to P14, P15 and P16 |
| *DMPK* | Pt14 | 9/>150 | Male | European | Hereditary motor and sensory neuropathy | Myotonia atrophica and neuropathy. Affected brother. 50y at examination | **YES** | Related to P13, P15 and P16 |
| *DMPK* | Pt15 | 8/>150 | Male | European | Hereditary motor and sensory neuropathy | Myotonia atrophica. Affected nephew. 35y at examination | **YES** | Related to P13, P14 and P16 |
| *DMPK* | Pt16 | 9/>150 | Female | European | Hereditary motor and sensory neuropathy | Myotonia atrophica. Affected nice. 29y at examination | **YES** | Related to P13, P14 and P16 |
| *DMPK* | Pt17 | 11/>100 | Female | European | Ataxia | Neurology outpatient for vestibular impairment and mental health. | No | Clinical diagnosis of myotonic dystrophy not confirmed, possibly due to age of proband below the age of onset |
| *DMPK* | Pt18 | 12/>150 | Female | European | Muscle channelopahty | IBM genetic study | No | Insufficient details |

| | | | | | | | | |
|---|---|---|---|---|---|---|---|---|
| *DMPK* | Pt19 | 12/60 | Male | Unknown | Hereditary motor and sensory neuropathy | Hereditary peripheral neuropathy. 42y at examination. | **YES** | |
| *DMPK* | Pt20 | 12/>100 | Male | European | Dementia | Hxof learning disabilities causing severe cognitive impairment which is progressive. Progressive accumulation of white matter in the brain. | No | Clinical diagnosis of myotonic dystrophy not confirmed, possibly due to age of proband below the age of onset |
| *HTT* | Pt21 | 29/53 | Female | European | Neurodevelopmental delay | Ataxia, hyperreflexia, chorea, no cognitive decline, no extra-ocular or sphincter involvement, cerebellar and brain stem atrophy on MRI. | **YES** | |
| *PPP2R2B* | Pt22 | Sep-34 | Male | European | Hereditary spastic paraplegia | Complex HSP | No | Reduced Penetrance |
| *PPP2R2B* | Pt23 | Sep-34 | Female | Unknown | Neurodevelopmental delay | Unaffected mother of Pt23 | No | Reduced Penetrance |
| *TBP* | Pt24 | 39/45 | Male | European | Dystonia | Reduced Penetrance | No | |
| *TBP* | Pt25 | 38/45 | Male | European | Dystonia | Reduced Penetrance | No | |
| *TBP* | Pt26 | 38/44 | Female | European | Epilepsy | Reduced Penetrance | No | |
| *TBP* | Pt27 | 38/57 | Male | Unknown | Ataxia | SCA familial and early death. 16 years old. Onset 13. Gait instability, dysarthria, truncal ataxia, limb ataxia, weakness. 7 other affected in 5 generation family | **YES** | |
| *TBP* | Pt28 | 40/44 | Male | South Asian | Neurodevelopmental delay | Onset 16 year old. Examined at age 18, gait ataxia, peripheral neuropathy, dysarthria, truncal ataxia, limb ataxia, weakness in the legs, legs, skeletal muscle atrophy, Babinski, lower limbs sensory loss, thoracic scoliosis | YES | |
| *TBP* | Pt29 | 39/45 | Female | European | Neurodevelopmental delay | Reduced Penetrance | No | Reduced Penetrance |

**Table S5.** Genotyping rate per coding loci, by genomic region and sequencing kit.

| Gene | LowDepth | PASS | Total |
|---|---|---|---|
| *AR* | 11.52% | 88.48% | 100.00% |
| Nextera DNA Focused | 13.38% | 86.62% | 100.00% |
| SureSelect V4 | 14.05% | 85.95% | 100.00% |
| SureSelect V6 | 5.52% | 94.48% | 100.00% |
| Truseq Exome targeted | 15.75% | 84.25% | 100.00% |
| *ATN1* | 0.98% | 99.02% | 100.00% |
| Nextera DNA Focused | 1.18% | 98.82% | 100.00% |
| SureSelect V4 | 0.16% | 99.84% | 100.00% |
| SureSelect V6 | 0.00% | 100.00% | 100.00% |
| Truseq Exome targeted | 8.15% | 91.85% | 100.00% |

| | | | |
|---|---|---|---|
| *ATXN1* | **1.87%** | **98.13%** | **100.00%** |
| Nextera DNA Focused | 2.06% | 97.94% | 100.00% |
| SureSelect V4 | 0.66% | 99.34% | 100.00% |
| SureSelect V6 | 0.96% | 99.04% | 100.00% |
| Truseq Exome targeted | 10.91% | 89.09% | 100.00% |
| *ATXN2* | **97.67%** | **2.33%** | **100.00%** |
| Nextera DNA Focused | 96.03% | 3.97% | 100.00% |
| SureSelect V4 | 97.88% | 2.12% | 100.00% |
| SureSelect V6 | 99.87% | 0.13% | 100.00% |
| Truseq Exome targeted | 91.16% | 8.84% | 100.00% |
| *ATXN3* | **10.89%** | **89.11%** | **100.00%** |
| Nextera DNA Focused | 24.85% | 75.15% | 100.00% |
| SureSelect V4 | 1.49% | 98.51% | 100.00% |
| SureSelect V6 | 2.39% | 97.61% | 100.00% |
| Truseq Exome targeted | 73.76% | 26.24% | 100.00% |
| *ATXN7* | **58.75%** | **41.25%** | **100.00%** |
| Nextera DNA Focused | 97.79% | 2.21% | 100.00% |
| SureSelect V4 | 71.03% | 28.97% | 100.00% |
| SureSelect V6 | 14.16% | 85.84% | 100.00% |
| Truseq Exome targeted | 100.00% | 0.00% | 100.00% |
| *CACNA1A* | **64.23%** | **35.77%** | **100.00%** |
| Nextera DNA Focused | 85.88% | 14.12% | 100.00% |
| SureSelect V4 | 71.32% | 28.68% | 100.00% |
| SureSelect V6 | 44.53% | 55.47% | 100.00% |
| Truseq Exome targeted | 69.61% | 30.39% | 100.00% |
| *HTT* | **81.73%** | **18.27%** | **100.00%** |
| Nextera DNA Focused | 98.82% | 1.18% | 100.00% |
| SureSelect V4 | 99.73% | 0.27% | 100.00% |
| SureSelect V6 | 41.79% | 58.21% | 100.00% |
| Truseq Exome targeted | 99.03% | 0.97% | 100.00% |
| *PABPN1* | **67.21%** | **32.79%** | **100.00%** |
| Nextera DNA Focused | 98.53% | 1.47% | 100.00% |
| SureSelect V4 | 98.51% | 1.49% | 100.00% |
| SureSelect V6 | 0.09% | 99.91% | 100.00% |
| Truseq Exome targeted | 88.40% | 11.60% | 100.00% |
| *PHOX2B* | **60.52%** | **39.48%** | **100.00%** |
| Nextera DNA Focused | 98.09% | 1.91% | 100.00% |
| SureSelect V4 | 79.37% | 20.63% | 100.00% |
| SureSelect V6 | 6.21% | 93.79% | 100.00% |
| Truseq Exome targeted | 99.86% | 0.14% | 100.00% |
| *TBP* | **3.51%** | **96.49%** | **100.00%** |
| Nextera DNA Focused | 19.41% | 80.59% | 100.00% |
| SureSelect V4 | 0.11% | 99.89% | 100.00% |

| | | | |
|---|---|---|---|
| SureSelect V6 | 0.00% | 100.00% | 100.00% |
| Truseq Exome targeted | 17.40% | 82.60% | 100.00% |
| **Grand Total** | **41.72%** | **58.28%** | **100.00%** |

**Table S6.** Genotyping rate per intronic loci, by genomic region and sequencing kit.

| Locus | LowDepth | PASS | Total |
|---|---|---|---|
| ***ATXN10*** | **100.00%** | **0.00%** | **100.00%** |
| Nextera DNA Focused | 100.00% | 0.00% | 100.00% |
| SureSelect V4 | 100.00% | 0.00% | 100.00% |
| SureSelect V6 | 100.00% | 0.00% | 100.00% |
| Truseq Exome targeted | 100.00% | 0.00% | 100.00% |
| ***C9ORF72*** | **100.00%** | **0.00%** | **100.00%** |
| Nextera DNA Focused | 100.00% | 0.00% | 100.00% |
| SureSelect V4 | 100.00% | 0.00% | 100.00% |
| SureSelect V6 | 100.00% | 0.00% | 100.00% |
| Truseq Exome targeted | 100.00% | 0.00% | 100.00% |
| ***CNBP*** | **99.97%** | **0.03%** | **100.00%** |
| Nextera DNA Focused | 100.00% | 0.00% | 100.00% |
| SureSelect V4 | 100.00% | 0.00% | 100.00% |
| SureSelect V6 | 100.00% | 0.00% | 100.00% |
| Truseq Exome targeted | 99.72% | 0.28% | 100.00% |
| ***CSTB*** | **98.84%** | **1.16%** | **100.00%** |
| Nextera DNA Focused | 99.85% | 0.15% | 100.00% |
| SureSelect V4 | 98.17% | 1.83% | 100.00% |
| SureSelect V6 | 99.52% | 0.48% | 100.00% |
| Truseq Exome targeted | 99.17% | 0.83% | 100.00% |
| ***FXN*** | **100.00%** | **0.00%** | **100.00%** |
| Nextera DNA Focused | 100.00% | 0.00% | 100.00% |
| SureSelect V4 | 100.00% | 0.00% | 100.00% |
| SureSelect V6 | 100.00% | 0.00% | 100.00% |
| Truseq Exome targeted | 100.00% | 0.00% | 100.00% |
| ***NOP56*** | **12.04%** | **87.96%** | **100.00%** |
| Nextera DNA Focused | 69.85% | 30.15% | 100.00% |
| SureSelect V4 | 1.12% | 98.88% | 100.00% |
| SureSelect V6 | 1.69% | 98.31% | 100.00% |
| Truseq Exome targeted | 47.51% | 52.49% | 100.00% |
| ***RFC1*** | **99.99%** | **0.01%** | **100.00%** |
| Nextera DNA Focused | 100.00% | 0.00% | 100.00% |
| SureSelect V4 | 100.00% | 0.00% | 100.00% |
| SureSelect V6 | 100.00% | 0.00% | 100.00% |

| | | | |
|---|---|---|---|
| Truseq Exome targeted | 99.86% | 0.14% | 100.00% |
| Grand Total | 87.26% | 12.74% | 100.00% |

**Table S7.** Genotyping rate per UTR loci, by genomic region and sequencing kit.

| Locus | LowDepth | PASS | Total |
|---|---|---|---|
| *ATXN8OS* | 98.45% | 1.55% | 100.00% |
| Nextera DNA Focused | 94.56% | 5.44% | 100.00% |
| SureSelect V4 | 100.00% | 0.00% | 100.00% |
| SureSelect V6 | 100.00% | 0.00% | 100.00% |
| Truseq Exome targeted | 89.09% | 10.91% | 100.00% |
| *CBL* | 79.80% | 20.20% | 100.00% |
| Nextera DNA Focused | 99.85% | 0.15% | 100.00% |
| SureSelect V4 | 71.46% | 28.54% | 100.00% |
| SureSelect V6 | 82.02% | 17.98% | 100.00% |
| Truseq Exome targeted | 97.38% | 2.62% | 100.00% |
| *DMPK* | 31.13% | 68.87% | 100.00% |
| Nextera DNA Focused | 67.79% | 32.21% | 100.00% |
| SureSelect V4 | 26.21% | 73.79% | 100.00% |
| SureSelect V6 | 31.41% | 68.59% | 100.00% |
| Truseq Exome targeted | 21.41% | 78.59% | 100.00% |
| *FMR1* | 99.96% | 0.04% | 100.00% |
| Nextera DNA Focused | 99.56% | 0.44% | 100.00% |
| SureSelect V4 | 100.00% | 0.00% | 100.00% |
| SureSelect V6 | 100.00% | 0.00% | 100.00% |
| Truseq Exome targeted | 100.00% | 0.00% | 100.00% |
| *GLS* | 40.83% | 59.17% | 100.00% |
| Nextera DNA Focused | 91.62% | 8.38% | 100.00% |
| SureSelect V4 | 2.84% | 97.16% | 100.00% |
| SureSelect V6 | 69.55% | 30.45% | 100.00% |
| Truseq Exome targeted | 99.45% | 0.55% | 100.00% |
| *JPH3* | 12.94% | 87.05% | 100.00% |
| Nextera DNA Focused | 78.38% | 21.62% | 100.00% |
| SureSelect V4 | 1.57% | 98.43% | 100.00% |
| SureSelect V6 | 0.00% | 100.00% | 100.00% |
| Truseq Exome targeted | 51.80% | 48.20% | 100.00% |
| *NIPA1* | 76.91% | 23.09% | 100.00% |
| Nextera DNA Focused | 99.41% | 0.59% | 100.00% |
| SureSelect V4 | 99.60% | 0.40% | 100.00% |
| SureSelect V6 | 25.89% | 74.11% | 100.00% |
| Truseq Exome targeted | 100.00% | 0.00% | 100.00% |

| | | | |
|---|---|---|---|
| *NOTCH2NLC* | 74.18% | 25.82% | 100.00% |
| Nextera DNA Focused | 96.32% | 3.68% | 100.00% |
| SureSelect V4 | 99.92% | 0.08% | 100.00% |
| SureSelect V6 | 24.20% | 75.80% | 100.00% |
| Truseq Exome targeted | 78.45% | 21.55% | 100.00% |
| *PPP2R2B* | 36.26% | 63.74% | 100.00% |
| Nextera DNA Focused | 91.32% | 8.68% | 100.00% |
| SureSelect V4 | 31.78% | 68.22% | 100.00% |
| SureSelect V6 | 15.33% | 84.67% | 100.00% |
| Truseq Exome targeted | 74.31% | 25.69% | 100.00% |
| Grand Total | 61.16% | 38.84% | 100.00% |
